# Chronic postoperative pain: recent findings in understanding and management

**DOI:** 10.12688/f1000research.11101.1

**Published:** 2017-07-04

**Authors:** Darin Correll

**Affiliations:** 1 Department of Anesthesiology, Perioperative and Pain Medicine, Brigham and Women’s Hospital, Boston, MA, USA; 2 Harvard Medical School, Boston, MA, USA

**Keywords:** persistent postoperative pain, surgery, multimodal analgesia

## Abstract

Chronic postoperative pain is a poorly recognized potential outcome from surgery. It affects millions of patients every year, with pain lasting for months to years, resulting in patient suffering and ensuing economic consequences. The operations with the highest incidence of chronic postoperative pain are amputations, thoracotomies, cardiac surgery, and breast surgery. Other risk factors include preoperative pain, psychological factors, demographics, and the intensity of acute postoperative pain. Attempts to prevent chronic postoperative pain have often led to debatable results. This article presents data from recently published studies examining the incidence, risk factors, mechanisms, treatment options, and preventive strategies for chronic postoperative pain in adults. In summary, many of the previously identified risk factors for chronic postoperative pain have been confirmed and some novel ones discovered, such as the importance of the trajectory of acute pain and the fact that catastrophizing may not always be predictive. The incidence of chronic postoperative pain hasn’t changed over time, and there is limited new information regarding an effective preventive therapy. For example, pregabalin may actually cause more harm in certain surgeries. Further research is needed to demonstrate whether multimodal analgesic techniques have the best chance of significantly reducing the incidence of chronic postoperative pain and to determine which combination of agents is best for given surgical types and different patient populations.

## Introduction

Chronic (or persistent) postoperative pain (CPOP) is a potentially devastating outcome from an otherwise successful surgical procedure. Patients experience pain (2–10% of the time of severe intensity ^1^) long after they have healed from the surgical insult. Chronic pain leads to increased direct medical costs by additional resource utilization and increased indirect costs through job absenteeism and loss of productivity. There is also a significant impact on individuals and their families, affecting their physical functioning, psychological state, and quality of life ^2^. The first publication that identified prior surgery as a cause of chronic pain came from a pain clinic in Northern England in 1998 where Crombie *et al*. found that almost one in four patients attributed their pain to an operation ^3^. Since that time, it has been shown that depending on the type of surgery, the incidence of CPOP is anywhere between 5 and 85% ^4^.

The criteria to establish a diagnosis of CPOP, proposed by Macrae and Davies in 1999, are 1) the pain must have developed after surgery, 2) the pain is of at least 2 months in duration, 3) other causes for the pain have been excluded, and 4) the possibility that the pain is continuing from a pre-existing problem must be explored and exclusion attempted ^5^. Unfortunately, many studies do not use all of these criteria; thus, being able to determine a “true” incidence for any given surgery is impossible. However, patterns have emerged and the surgeries with the highest incidence of CPOP have been shown to be amputations (50–85%), thoracotomies (5–65%), cardiac surgery (30–55%), and breast surgery (20–50%) ^4^. There has also been a recent suggestion by Werner and Kongsgaard to change the criteria for the diagnosis of CPOP based on current understanding ^6^. The newly proposed criteria are as follows: 1) the pain develops after a surgical procedure or increases in intensity after the surgical procedure, 2) the pain is of at least 3–6 months’ duration and significantly affects quality of life, 3) the pain is a continuation of acute post-surgery pain or develops after an asymptomatic period, 4) the pain is localized to the surgical field, projected to the innervation territory of a nerve situated in the surgical field, or referred to a dermatome, and 5) other causes of the pain should be excluded ^6^.

In addition to the type of surgery, there are numerous other risk factors for the development of CPOP that have been identified, including having preoperative pain, psychological factors (e.g. anxiety, depression, and catastrophizing), demographics (e.g. female gender and younger age), surgical factors (e.g. open approach and length >3 hours), and the intensity of pain in the immediate postoperative period (i.e. first few days). Given recent estimates for the number of surgeries performed every year across the world and using a conservative estimate of 10% for the overall incidence of CPOP ^2^, that would mean that approximately 23 million people per year are afflicted with this awful problem.

This review will summarize data from some of the most recently published (January 2014 – February 2017) clinical adult human studies and meta-analyses examining CPOP that address the topics of incidence, risk factors, mechanisms, treatment options, and preventive strategies. It will not address genetic analyses nor will it discuss results from animal, pediatric, experimentally induced, obstetric, or dental pain studies. The search terms “chronic postoperative pain”, “chronic postsurgical pain”, “chronic post- pain”, “persistent postoperative pain”, “persistent postsurgical pain”, and “persistent post- pain” were entered into the Cochrane Central Register of Controlled Trials (CENTRAL, issue 11, 2016), PubMed, and the Cochrane Database of Systematic Reviews to generate the list of potential articles. In addition, a hand search of other reviews from the stated time-frame was performed. Titles and abstracts were examined to remove obviously irrelevant reports.

The studies from the last few years show that, unfortunately, the incidence of CPOP hasn't changed over time. These recent publications confirm many of the previously known risk factors and identify a few novel ones. The exact mechanisms of CPOP remain uncertain, and the treatment of established CPOP continues to be a challenge. Finally, there is limited new information on an effective pharmacological preventive therapy; in fact, much of the recent information has demonstrated what does not work as opposed to what does.

## Incidence and risk factors

In 2015, Fletcher *et al*. conducted a survey of CPOP at 21 hospitals across 11 European countries and found, at 6 months, the overall incidence of mild pain (score = 1–2/10) to be 24% and of moderate-to-severe pain (score ≥ 3/10) to be 16%; at 12 months, the incidence of mild pain was 23% and of moderate-to-severe pain was 12% ^2^. Acknowledging a very low follow-up response rate (≈30%) in this study, the authors identified a novel risk factor that deserved further investigation. The duration of severe pain in the initial 24 hours postoperatively, as opposed to the intensity of pain, predicted the chance of developing CPOP ^2^. For every 10% increase in time spent in severe pain, the risk of developing CPOP went up by 30% ^2^. Also in 2015, a study of outpatient surgeries found an overall incidence of CPOP of 15% at 1 year ^7^. The risk factors determined were surgical specialty (i.e. urology, general, plastic, and orthopedic), preoperative pain, preoperative analgesic use, acute postoperative pain, surgical fear, lack of optimism, and poor preoperative quality of life ^7^. A 2017 analysis of prior studies examined the association between acute pain intensity and CPOP following a number of different surgeries ^8^. The results suggest that one reason for the variability in the observed association between acute pain and CPOP in the literature may be the method of measurement of acute pain (i.e. at rest versus after movement) ^8^. The remaining recent studies on the incidence and risk factors for CPOP examine specific surgical procedures.

### Abdominal surgery

In 2014, Holtzman *et al*. studied liver donation patients and found a CPOP incidence of 31% and 27% at 6 and 12 months, respectively, identifying female sex, younger age, and preoperative pain-related anxiety as risk factors ^9^. A 2015 retrospective analysis found a CPOP incidence after laparoscopic colorectal surgery of 17% (similar to open surgery) and determined the risks as redo surgery for anastomotic leakage, inflammatory bowel disease, and preoperative pain ^10^. Using a retrospective questionnaire in 2016, Jeppesen *et al*. determined that the incidence of CPOP following emergency laparotomy for bowel obstruction was 21% ^11^. Also in 2016, a study of women undergoing abdominally based autologous breast reconstruction found a CPOP incidence of 24% and 23% at 6 and 12 months, respectively, with those reporting greater variability, at rest, between maximum and minimum pain levels over the first 48 hours postoperatively having a higher risk of developing CPOP at 6 months ^12^.

### Breast surgery

The latest studies have shown a CPOP incidence of 30–60% ^13– 16^ with the incidence of moderate-to-severe pain being 14% ^17^. In 2014, a retrospective study showed no difference in CPOP incidence if patients had breast reconstruction or mastectomy alone ^14^, whereas, in 2015, a prospective trial found that breast implant, as well as adjuvant radiotherapy, did increase the likelihood of CPOP development ^13^. Also in 2015, Mejdahl *et al*. showed that preoperative distress is a risk factor for CPOP ^18^, and Andersen *et al*. found the risk factors to be <65 years of age, breast-conserving surgery, axillary lymph node dissection, moderate-to-severe preoperative pain, acute postoperative pain, lower preoperative diastolic blood pressure, and signs of neuropathic pain at 1 week ^17^. In 2016, Baudic *et al*. established the presence of alexithymia (the inability to identify and express emotions) as a novel risk factor for CPOP ^19^. They also found that catastrophizing was predictive of pain only until 2 months postoperatively, suggesting it is important in the transition from subacute pain to CPOP but not necessarily its maintenance ^19^. Also in 2016, a retrospective questionnaire found low income, presence of post-traumatic stress disorder (PTSD), and <46 months from the time of surgery as risks for CPOP after mastectomy ^15^, and a meta-analysis established the CPOP risks as younger age, radiotherapy, axillary lymph node dissection, acute postoperative pain, and preoperative pain ^20^.

### Cardiac surgery

Recent studies in patients following sternotomy found the incidence of CPOP at 3 months to be 43% ^21^ but only 11% at 12 months and 3.8% at 5 years ^22^. In 2016, Setälä *et al*. demonstrated that the area of hyperalgesia around the sternotomy wound, on postoperative day 4, was not associated with CPOP at 4–6 months ^23^, and Guimarães-Pereira *et al*. found the risk factors to be younger age, female gender, higher body mass index, catastrophizing, coronary artery bypass graft, osteoarthritis, history of previous surgery, and moderate-to-severe acute postoperative pain ^21^.

### Hysterectomy

Two studies from the past few years, both for benign causes, found an overall incidence of CPOP of 26% at 6 months after laparoscopic or vaginal hysterectomy ^24^ and an incidence of moderate-to-severe CPOP of 10.2% and 9.0%, respectively, at 3 and 12 months after vaginal, abdominal, or laparoscopic hysterectomy ^25^. The 2015 study of laparoscopic or vaginal hysterectomy found risk factors to be a history of smoking, severe pain 4 hours after surgery, and laparoscopic approach ^24^, whereas the 2016 study of vaginal, abdominal, or laparoscopic hysterectomy determined the risks to be moderate-to-severe preoperative pain, surgery-related worries, postoperative pain on day 4, and surgery-related infection ^25^.

### Inguinal hernia surgery

A 2015 trial of unilateral or bilateral, primary or recurrent, inguinal hernia surgery found that at 1 year the incidence of CPOP was 43% for patients aged 18–40 years old, 29% for patients aged 40–60 years old, and only 19% for patients >60 years old ^26^. Bugada *et al*., in 2016, studied unilateral open inguinal hernia repair with mesh and found a CPOP incidence of 9.3% at 3 months and that the intensity of CPOP was correlated with preoperative arterial hypertension ^27^. A 2016 registry analysis of unilateral first and second recurrent inguinal hernia repairs showed an incidence of CPOP of 29% (a median of 4½ years after surgery) and that anterior mesh repair (compared to endoscopic posterior mesh repair) or a second recurrent repair resulted in a higher incidence of CPOP ^28^.

### Total knee arthroplasty

Recent studies of osteoarthritis patients found a CPOP incidence of 58% (11% of those having a neuropathic component) ^29^ with an incidence of moderate-to-severe CPOP of 22% ^30^. A 2014 study found that patients with severe pain during a simple knee active flexion and extension test prior to total knee arthroplasty (TKA) had a 10-times higher likelihood of moderate-to-severe pain at 6 months ^31^. Also in 2014, Lavand'homme *et al*. found the predictors for neuropathic pain to be a stable trajectory for maximal pain and a rising trajectory for pain during mobilization over the first 8 days postoperatively compared to downward trajectories seen in patients without neuropathic pain ^29^. However, in 2015, Pagé *et al*. found that the predictor for more intense CPOP at 12 months after TKA was a moderate level of preoperative pain which resulted in a flat to only slightly rising pain trajectory over the first 6 weeks postoperatively with a rise from 3 to 12 months ^32^. This was in contrast to having either a high level of preoperative pain (which resulted in either a constant downward pain trajectory to 12 months or a flat trajectory to 3 months before a decline to 12 months) or a low level of preoperative pain (which resulted in an overall downward trajectory to 12 months) ^32^. In addition, they failed to show that age or gender was a risk factor for CPOP ^32^. A 2015 study of female osteoarthritis patients (with a CPOP incidence of 16.5% at 3 months) found the strongest determinant of CPOP was a high preoperative central sensitization inventory score, which is a measure that assesses 25 somatic and emotional symptoms, suggesting the presence of central sensitization (it has not been validated in TKA, but the results are promising) ^33^. Another 2015 study of osteoarthritis patients evaluated the predictive value of three types of preoperative quantitative sensory tests (i.e. pressure pain thresholds, temporal summation of pain, and conditioned pain modulation) and found that only temporal summation correlated with CPOP at 1 year ^30^. A 2015 meta-analysis by Lewis *et al*., of patients 3 months to 7 years following TKA (unilateral or bilateral for osteoarthritis or rheumatoid arthritis), found that the strongest predictors of CPOP were mental health, preoperative knee pain, pain at other sites, and catastrophizing ^34^. To the contrary, a 2016 prospective cohort study found no association between catastrophizing and CPOP at 2 months or 1 year ^35^. Furthermore, they found a large reduction in catastrophizing scores from baseline to follow-up ^35^.

### Other orthopedic surgery

A 2015 questionnaire, 1–2 years after shoulder replacement, found a CPOP incidence of 22% (29% in fractures and 16% in osteoarthritis) with an incidence of presumed neuropathic pain of 13% ^36^. Risk factors for CPOP were severe pain during the first postoperative week, use of a hemiprosthesis in osteoarthritis patients (as compared to a total prosthesis), and a history of previous fracture fixation and pain elsewhere in fracture patients ^36^. In 2015, Coronado *et al*. studied chronic pain patients undergoing lumbar laminectomy and determined that pain sensitivity at 6 weeks postoperatively (as measured by pressure pain threshold) predicted the intensity of CPOP at 6 months, while pain catastrophizing predicted not only pain intensity but also the degree of pain interference and disability ^37^. A 2016 questionnaire, 1 year after ankle or wrist fracture surgery, found a CPOP incidence of 19% (of these, 43% had presumed neuropathic pain) ^38^.

### Thoracic surgery

All of the recent thoracotomy studies show a similar CPOP incidence: 57% at 3 months ^39^, 39–56% at 6 months ^39– 41^, and 50% at 1 year ^42^. A 2014 meta-analysis by Bayman and Brennan determined that the incidence has been stable from the 1990s until the time of their analysis ^39^. Preoperative pain is consistently shown to be a predominant predictor for CPOP ^40, 42^, as well as poor pain control during the first 72 hours after surgery ^41^, while "dispositional optimism" is protective against it ^42^. In 2016, Wildgaard *et al*. showed that video-assisted thoracic surgery has a lower incidence of CPOP (11–30%, depending on the definition) compared to open thoracotomies ^43^.

### Thyroidectomy

A 2016 study found a 37% incidence of CPOP at 3 months following robot-assisted thyroidectomy ^44^. Another 2016 study found the incidence of neuropathic pain after thyroidectomy to be 12% at 3 months and 9% at 6 months ^45^. Risk factors for the development of CPOP were found to be preoperative anxiety and the need for preoperative information ^45^.

## Neural-based mechanisms

Damage to major nerves during surgery is an important causal factor in most cases of CPOP; however, not all patients who have nerves injured go on to develop chronic pain, and pain can occur in the absence of known nerve sectioning ^4^. Much CPOP has been shown to be neuropathic in nature. For example, in 2016, it was found that patients who developed CPOP after breast cancer surgery were significantly more hyperalgesic to generalized pressure both 5 days after surgery and from 15 days to 12 months compared to patients who did not develop pain, suggesting that persistent central sensitization is an important process in CPOP development ^46^. Also in 2016, a trial of 14 patients with CPOP, following inguinal hernia surgery, found that a tender point block with bupivacaine provided analgesia and reduced evoked pain responses, supporting the role of peripheral afferents in the maintenance of pain ^47^. In addition to central sensitization from the surgical insult, which is maintained by peripheral input and peripheral sensitization of the primary nociceptors in the surgical field, other (non-mutually exclusive) potentially causal mechanisms for CPOP have been suggested ^48^. These include intraoperative nerve damage with resultant injury discharge, early postoperative ectopic activity in the regenerating sprouts of the damaged primary afferents and in the cell bodies of these injured nerve fibers in the dorsal root ganglia (or even in the cell bodies of intact neurons adjacent to those associated with the injured nerves), collateral sprouting from undamaged nociceptive (A delta) afferents that are adjacent to the field innervated by the injured afferents, or structural changes in the central nervous system (e.g. disappearance of anti-nociceptive inhibitory interneurons in the dorsal horn caused by continued perioperative nociceptive input) ^48^. It is vital to determine the mechanism of the pain generator so that the correct agents or interventions can be chosen for treatment or prevention.

## Treatment

The treatment of chronic pain, in general, is difficult, and this certainly includes CPOP. Only a few recent studies have looked at CPOP treatment. A 2014 study of patients with CPOP following inguinal hernia repair (with a median duration of pain of 1½ years) found that an 8% capsaicin patch did not significantly reduce static or dynamic pain at 1–3 months after patch application ^49^. In 2016, a small (n=6) pilot study in women with CPOP in the axilla, upper arm, or lateral chest wall between 1 and 7 years after breast surgery demonstrated at least a mild analgesic effect following local anesthetic blockade of the intercostobrachial nerve that lasted for 7 days in half of the patients ^50^. In 2017, another small (n=8) pilot study in women with CPOP between 7 and 84 months after breast surgery demonstrated an analgesic effect that lasted for 7 days following local anesthetic blockade of the pectoral nerves ^51^. While promising, both of these regional techniques require much larger, randomized controlled trials to determine their true benefits.

## Prevention

Given that the treatment of already established CPOP is difficult, it has been suggested that attempts at prevention are crucial ^52^. Likely, one must achieve effective early postoperative analgesia and then continue it for as long as the nociceptive input is in a pathologic range (i.e. causing sensitization). It has been previously suggested that the choice of agents must include those that prevent sensitization ^1^ (e.g. gabapentinoids or N-methyl-D-aspartate receptor antagonists) and not just treatment of somatic pain (e.g. opioids). For example, a 2014 meta-analysis of varying surgical procedures showed that intravenous ketamine (in a range of doses, both bolus only and bolus plus a subsequent infusion) produces a 25% reduction (number needed to treat [NNT] of 12) in the risk of CPOP at 3 months and reduces the risk by 30% (NNT of 14) at 6 months but has no effect on risk reduction at 1 year ^53^. Unfortunately, given that the degree of relative risk reduction is small and considering the variable timing and dosing of ketamine in these studies, there is no specific regimen that can be suggested ^53^. On the other hand, a 2017 meta-analysis of varying surgical procedures found that pregabalin has no impact on the incidence of CPOP at 3, 6, or 12 months after surgery ^54^. Anti-inflammatory agents might be of benefit, since, as stated previously, not all CPOP is neuropathic in nature and inflammation can contribute to its generation and maintenance. Other recent studies on the prevention of CPOP have been conducted in specific surgical procedures.

### Abdominal surgery

A 2016 study of women undergoing abdominally based autologous breast reconstruction found no reduction in the incidence of CPOP from a 3-day bilateral transversus abdominis plane (TAP) infusion ^12^. A 2016 nephrectomy study found no difference in the incidence of CPOP between a laparoscopic versus open approach ^55^. In 2017, Capdevila *et al*. found that, after open nephrectomies, a continuous wound local anesthetic infusion resulted in a lower incidence of CPOP compared to both thoracic epidural and patient-controlled analgesia (PCA) morphine ^56^.

### Breast surgery

A 2015 study showed no reduction in CPOP from the perioperative use of 4–20 mg dexamethasone ^57^. However, another 2015 trial, by Terkawi *et al*., showed that intravenous lidocaine (a bolus at induction followed by an infusion up to 2 hours after surgery) decreased the incidence of CPOP ^13^. A 2015 meta-analysis concluded that thoracic paravertebral blocks may reduce the incidence of CPOP but cautioned that the evidence was severely limited ^58^. Then, in 2016, another meta-analysis was unable to confirm or disprove that paravertebral blocks can prevent CPOP development ^59^. Also in 2016, van Helmond *et al*. found that adding perioperative cyclooxygenase (COX)-2 inhibition to a ** paravertebral block has no significant impact on CPOP ^46^, whereas Na *et al*. showed that 20 mg intravenous nefopam preoperatively reduced CPOP incidence ^16^.

### Cardiac surgery

A 2015 trial showed that 500 mg intraoperative methylprednisolone did not alter the incidence of CPOP following on-pump cardiac surgery via a sternotomy ^60^. Another 2015 trial, of robotic surgery performed through a thoracotomy, failed to show a reduction in CPOP from up to 6 months of 5% lidocaine transdermal patches ^61^.

### Total knee arthroplasty

In 2014, Aveline *et al*. studied osteoarthritis patients and showed no significant benefit on the incidence of CPOP from the addition of a 2-day infusion of nefopam or ketamine (to a multimodal regimen of opioid, acetaminophen, and NSAID), although ketamine demonstrated some reduction in the neuropathic component ^62^. Also in 2014, Clarke *et al*. studied osteoarthritis patients and showed no benefit on the incidence of CPOP from the addition of a 4-day perioperative course of gabapentin (600 mg preoperatively followed by 200 mg three times daily) to a multimodal regimen of opioid, celecoxib, spinal anesthesia, and femoral and sciatic nerve blocks ^63^. A 2015 study failed to show a reduction in the severity of pain 12 months after TKA from an intraoperative local anesthetic infiltration of 0.25% bupivacaine (when added to a femoral nerve block and a spinal or general anesthetic) ^64^.

### Total hip arthroplasty

This same 2015 study did show a statistically significant reduction in the severity of pain 12 months after total hip arthroplasty (THA) from an intraoperative local anesthetic infiltration of 0.25% bupivacaine (when added to a spinal anesthetic); however, the difference was likely not clinically significant ^64^. Another 2015 study found that perioperative administration of pregabalin (150 mg preoperatively followed by 75 mg twice daily until 1 week after discharge) did not improve the incidence or severity of pain at 6 weeks or 3 months after THA when added to a spinal anesthetic plus celecoxib ^65^.

### Inguinal hernia surgery

A 2015 study failed to demonstrate any reduction in CPOP development from local infiltration of 20 mL 0.25% bupivacaine ^66^, whereas, in 2016, Bugada *et al*. found that local infiltration or spinal anesthesia reduced CPOP incidence when patients also received postoperative ketorolac for 4 days ^27^.

### Thoracic surgery

In 2014, 48 hours of either epidural or intravenous ketamine, added to a postoperative epidural regimen, showed no reduction in the incidence of CPOP after thoracotomy ^67^. Also in 2014, another thoracotomy trial showed no reduction in the incidence of CPOP from the addition of gabapentin (from 2 hours before surgery until 5 days postoperatively) to a 3-day epidural regimen plus oral acetaminophen, ibuprofen, and morphine ^68^. Then, in 2015, Brulotte *et al*. showed that twice-daily 150 mg pregabalin started 1 hour before induction and continued for 4 days, in addition to a thoracic epidural, increased the incidence of CPOP after thoracotomy ^69^. However, the pregabalin group reported less severe average pain, required fewer analgesics, and had less neuropathic pain than did the placebo group ^69^. A 2016 trial of 300 mg pregabalin started 30 minutes before induction followed by 150 mg twice daily for 5 days showed a non-statistically significant increase in the incidence of CPOP after video-assisted thoracoscopic surgery ^70^, whereas a 2016 trial of 40 mg intravenous parecoxib started 30 minutes before induction and then every 12 hours for 3 days, in addition to a thoracic epidural, decreased the incidence of CPOP after thoracotomy and video-assisted thoracoscopic surgery ^71^.

### Thyroidectomy

In 2016, Wattier *et al*. demonstrated that having a bilateral superficial cervical plexus block in combination with a general anesthetic reduced the chance of CPOP after thyroidectomy ^45^. Also in 2016, Choi *et al*. showed that intravenous lidocaine (a bolus at induction followed by an infusion until extubation) decreased the incidence of CPOP after robot-assisted thyroidectomy ^44^.

## Summary

Table 1 lists the incidence from the latest studies of CPOP after various surgical procedures. Several of the most common previously described risk factors for CPOP are confirmed: female gender (in abdominal ^9^ and cardiac surgery ^21^), younger age (in abdominal ^9^, breast ^17, 20^, cardiac ^21^, and inguinal hernia surgery ^26^), preoperative pain (in abdominal ^10^, breast ^17, 20^, and outpatient surgery ^7^, hysterectomy ^25^, TKA ^34^, and thoracotomy ^40, 42^), psychological states (anxiety in abdominal surgery ^9^ and thyroidectomy ^45^, distress ^18^ and PTSD ^15^ in breast surgery, catastrophizing in cardiac surgery ^21^ and lumbar laminectomy ^37^, and fear and lack of optimism in outpatient surgery ^7^), and postoperative pain (in breast ^17, 20^, cardiac ^21^, shoulder replacement ^36^, and outpatient surgery ^7^, hysterectomy ^24, 25^, and thoracotomy ^41^). The duration of severe pain in the initial 24 hours postoperatively, as opposed to the pain intensity, predicts the chance of CPOP ^2^. An endoscopic approach for inguinal hernia surgery lowers the incidence of CPOP ^28^ but laparoscopic colorectal surgery does not ^10^. For breast surgery, other known risk factors (radiotherapy ^13, 20^ and axillary dissection ^17, 20^) were confirmed, and the novel risk of alexithymia was determined ^19^. In addition, studies are contradictory if breast reconstruction contributes to CPOP ^13, 14^, and the exact relevance of catastrophizing has been called into question ^19^. After TKA, an interesting risk factor for the intensity of CPOP is having moderate preoperative pain, as opposed to high or low, which results in different pain trajectories ^32^. Also, following TKA, another novel CPOP risk is the trajectory of pain over the first week as a predictor of neuropathic pain ^29^, and catastrophizing may not be correlated with CPOP ^34, 35^. In addition, it may be that prior evidence of catastrophizing remaining constant over time is not accurate ^35^.

**Table 1.** Chronic postoperative pain (CPOP) incidence by surgical procedure from recent studies.

Surgery	CPOP incidence	Moderate-to-severe CPOP incidence
Abdominal *	17–31% ^#^	-
Breast	30–60% ^‡^	14%
Cardiac	4–43% ^‡^	-
Hysterectomy	26%	9–10%
Inguinal hernia	9–43% ^#^	-
Orthopedic ^∇^	19–22%	-
Outpatient ^+^	15%	-
Total knee arthroplasty	16–58%	22%
Thoracotomy	39–57% ^#^	-
Video-assisted thoracoscopy	11–30%	-
Thyroidectomy	37%	-

* liver donation, laparoscopic colorectal, emergency laparotomy, and abdominally based autologous breast reconstruction ∇ shoulder replacement and ankle or wrist fracture repair + those with highest risk are urology, general, plastic, and orthopedic # no decrease in incidence over time ‡ decrease in incidence over time

TAP catheters or epidurals do not reduce CPOP development in certain abdominal surgeries ^12, 56^, and paravertebral blocks do not seem to be of benefit in breast surgery ^58, 59^; however, superficial cervical plexus blocks are beneficial for thyroidectomy ^45^. Intravenous lidocaine reduces CPOP following breast surgery ^13^ and thyroidectomy ^44^. Glucocorticoids show no CPOP reduction in either breast ^57^ or cardiac surgery ^60^. Nefopam reduces CPOP incidence after breast surgery ^16^ but fails to be effective after TKA ^62^. Local infiltration of bupivacaine does not provide a meaningful reduction in CPOP after inguinal hernia surgery ^66^, TKA ^64^, or THA ^64^. However, spinal or local infiltration anesthesia does reduce CPOP incidence following inguinal hernia repair in the presence of a COX inhibitor ^27^ and the addition of a COX inhibitor to an epidural reduces the incidence of CPOP following thoracic surgery ^71^, but, following breast surgery, a COX inhibitor added to a paravertebral block has no effect on CPOP ^46^. Ketamine appears to have a small relative risk reduction for CPOP development when various surgical procedures are analyzed together ^53^ but fails to show significant benefit after TKA specifically ^62^ or when added to an epidural regimen after thoracotomy ^67^. Gabapentin shows no added benefit on CPOP when added to a multimodal regimen of opioid, celecoxib, spinal anesthesia, and femoral and sciatic nerve blocks following TKA ^63^. Pregabalin shows no benefit on the incidence of CPOP when multiple surgical types are analyzed together ^54^ nor does it provide benefit when added to a spinal block plus celecoxib following THA ^65^. Adding pregabalin to an epidural regimen following thoracotomy ^69^ or using it after video-assisted thoracoscopic surgery ^70^ may actually make the incidence of CPOP worse.

## Remaining questions

Recent literature lacks studies examining the potential benefits of non-pharmacological methods for CPOP treatment or prevention (e.g. acupuncture, transcutaneous electrical nerve stimulation, etc.). Despite the fact that pain is a completely subjective experience and the risk for CPOP includes multiple emotional factors, no recent studies have investigated psychologically based therapies (e.g. cognitive-behavioral therapy, guided imagery, hypnosis, mindfulness, etc.). Given the fact that CPOP continues to be undermanaged, it would seem prudent to scientifically evaluate all possible options. In 2015, Katz *et al*. published an article that describes the development and implementation of a multidisciplinary “Transitional Pain Service” with the expressed purpose of preventing CPOP ^72^. An integral part of this service is the use of acceptance and commitment therapy (ACT), which is a type of cognitive behavioral therapy that incorporates mindfulness, acceptance, and an emphasis on behavioral choices based on personal values ^72^. Future randomized controlled trials on the benefits of this program will hopefully greatly add to the treatment options for CPOP.

In 2016, a survey-based study of 1,005 individuals presenting to a preoperative testing clinic showed that 80% of patients never had a discussion with any provider of the possibility of CPOP development and that 43% of people thought their risk was 10% or less (24% thought their chance was zero) ^73^. In addition, it was determined that 65% of patients wanted to know their risk and almost 30% said that having this knowledge might or definitely would affect their decision to proceed with surgery ^73^. This risk is not a part of standard consents despite the fact that it occurs more frequently than many of the other risks that are commonly discussed (e.g. major bleeding and infection). Thus, it is imperative that healthcare providers begin to discuss the significant risk of CPOP with patients prior to surgery. Patients need to be aware of their potential risk of CPOP development to ensure that their expectations align with possible outcomes and for them to be able to give proper informed consent.

The many negative effects from CPOP (including patient suffering and economic consequences) make this one of the most important outcome benefits that can be realized from postoperative pain management. Attempts at preventing CPOP with individual agents frequently lead to questionable results. Multimodal techniques will likely have the best chance of working, as two of the recent studies have shown ^27, 71^, but more research is needed to prove this and to determine which combinations are best for given surgical types and different patient populations. Unfortunately, it will be difficult to do the perfect, prospective, randomized, double-blind, placebo-controlled trial to prove the optimal regimen because what is necessary is a highly individualized approach based on the entire characteristics of each patient.

## Abbreviations

COX, cyclooxygenase; CPOP, chronic postoperative pain; NNT, number needed to treat; PTSD, post-traumatic stress disorder; TAP, transversus abdominis plane; THA, total hip arthroplasty; TKA, total knee arthroplasty.
